# The Next Step for Motor Proteins

**DOI:** 10.1371/journal.pbio.1001043

**Published:** 2011-04-12

**Authors:** Mason Inman

**Affiliations:** Freelance Science Writer, Berkeley, California, United States of America

**Figure:**
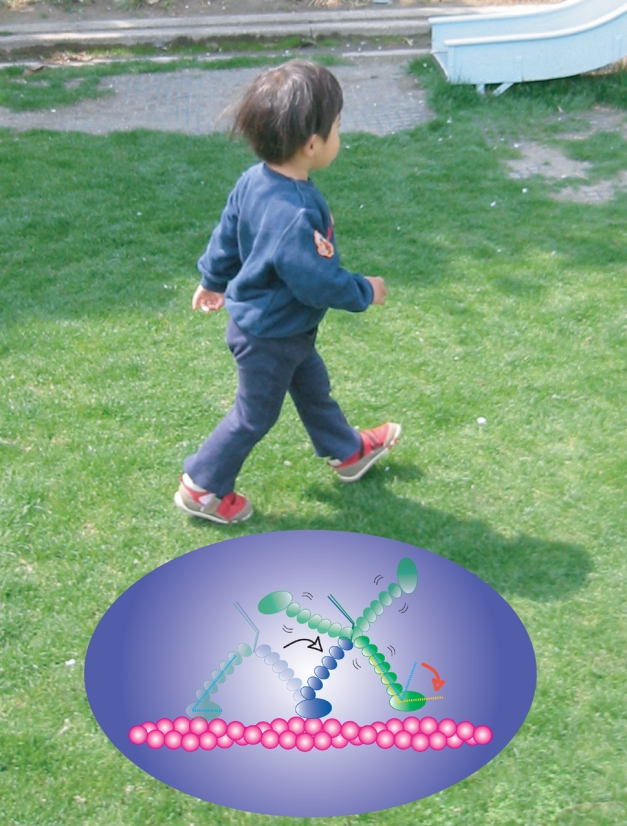
Myosin V points its “toe” stably down to step forward.

When you walk, as you go through each step, you naturally point and flex your foot. Your toes are the last part of your foot to lift off the ground as you finish a step, and when you start a new step, your heel is the first part to hit the ground.

When you walk backwards, though, it's the opposite: when you lift your feet off the ground, your heel is the last part of your foot touching the ground, and the toe is the first part to touch ground on a new step. If you try walking backwards, but flex your foot so your heel strikes first, you'll probably walk much slower and are liable to fall down.

It's much the same with a molecular motor known as myosin Va, according to a new study in *PLoS Biology* by Katsuyuki Shiroguchi of Harvard University and colleagues. The new study shows the way myosin bends its "ankle" before each step. This bending had been theorized before, but now it has been observed for the first time—and it explains, the new study argues, how myosin is able to keep stepping forward along actin filaments.

There are more than a dozen types of myosins, motor proteins that can move along the actin filaments that crisscross cells. Just as ants can carry crumbs much bigger than themselves, myosin can ferry loads much larger than itself, such as vesicles that contain wastes that cells want to dump, or signaling molecules for sending messages. Maintaining this regular traffic within the cell, and to the outside of the cell, is essential for normal development and functioning.

Each myosin molecule is shaped somewhat like a snake, with a large "head" and a long "neck" stretching out from the head. Shiroguchi et al. studied one common type of myosin, known as myosin Va, which normally exists as a dimer, with the two molecules' necks wrapped around each other. They resemble the medical symbol with two intertwined snakes, with their heads pointing in opposite directions.

The conventional names for the parts of the myosin molecule are a bit confusing for a molecule known for its ability to walk. Myosin's "head" is the part that grabs onto the actin filament, and the "neck" is the part that can flex, helping pull myosin along. Myosin moves along actin by grabbing on with one head, pulling itself forward, swinging the other head forward, and latching that head on a bit farther along the actin filament. It's somewhat like the pair of entwined snakes climbing a ladder by taking turns, with one snake grabbing a rung of the ladder in its mouth, then swinging the other snake upward so that it can bite the next rung in its mouth, and so on.

Myosin tends to only move in one direction along an actin filament, a crucial property for making sure its payloads reach their destinations. If myosin was equally likely to take a step forward as a step back, it would just wander back and forth on an actin filament and wouldn't make much progress. Thus, understanding how myosin always takes a step forward each time has been the focus of a lot of study among biophysicists and other researchers.

Studies have shown that one important part of the process is known as the "power stroke," in which myosin's neck leans forward, in the direction that the molecule is moving. To make this power stroke, myosin needs an input of energy, which is supplied by binding to adenosine triphosphate (ATP), the common energy carrier in cells, and breaking off one phosphate group to form adenosine diphosphate (ADP).

Studies of the power stroke haven't provided a full answer on how myosin Va steps forward each time—and the same distance each time. Shiroguchi and his colleagues, in a 2007 study, found that when one of myosin's heads releases from the actin to take a step forward, that head can flail around, battered by water molecules and others in its surroundings, in random, "Brownian" motion. Somehow the flailing head manages to grab onto the actin filament farther along it, in the direction of motion. And in myosin Va, it grabs hold a precise distance away from the other head—72 nanometers away, to be exact.

From earlier research, many researchers have recognized that there was another step involved, called the "recovery stroke," which primes myosin for the next step after the power stroke. It wasn't clear how the recovery stroke worked, however, but it was suspected that it involved a bend in the angle between the head and neck—similar to how you point and flex your foot as you walk.

Shiroguchi and colleagues devised a way to measure whether myosin is bending between its head and neck—and if so, how much. They took single myosin molecules (not dimers), and immobilized them by tying down their necks to a surface by binding them to small beads. Then they attached large beads onto the head of each myosin molecule, using an antibody to make the attachment. The team also suspected that for myosin to bend, it would require energy. So they used a type of "caged" ATP in which the molecular cage opens suddenly when hit with a burst of UV light, and the ATP is released and can be used.

With this set-up, they were able to expose myosin to ATP with precision timing. Watching it under a microscope, they could see whether ATP would trigger the myosin heads to waggle back and forth. They found the heads did move—flexing by about 90 degrees, relative to the neck, each time they were exposed to ATP. This process seemed to have been powered by the ATP, with it breaking apart into ADP and a phosphate group, both of which remained bound to the head.

They also found that when the head flexed in this way, it moved into a new stable state and remained in the new position for a long time, in molecular terms. They typically stayed in the new position for around 40 seconds—about 500 times longer than it takes for myosin Va to take each step.

Shiroguchi and colleagues argue that the stability of this flexed state is crucial for enabling myosin to step along actin filaments. By bending the head by a specific angle and locking it there, myosin then is "primed" to attach to actin farther down the filament. During each step along an actin filament, the unattached myosin head would flail about. But by moving the head into the flexed state, it "primed" myosin to make the next step. In the flexed state, the angle of the head is such that it would bind easily to actin farther down the filament, but not be able to bind easily on the filament in the backwards direction. It's much like how, by bending your foot back toward your leg, you prepare for a step forward, in which your heel strikes the ground first.

By observing this process, Shiroguchi et al. have resolved a key step in the movement of myosin. It helps make sense of this essential process, and could also aid in nanotechnology, since myosins and other similar proteins are being engineered to work as tiny motors and pumps in artificial systems.

**Shiroguchi K, Chin HF, Hannemann DE, Muneyuki E, De La Cruz EM, et al. (2011) Direct Observation of the Myosin Va Recovery Stroke That Contributes to Unidirectional Stepping along Actin. doi:10.1371/journal.pbio.1001031**

